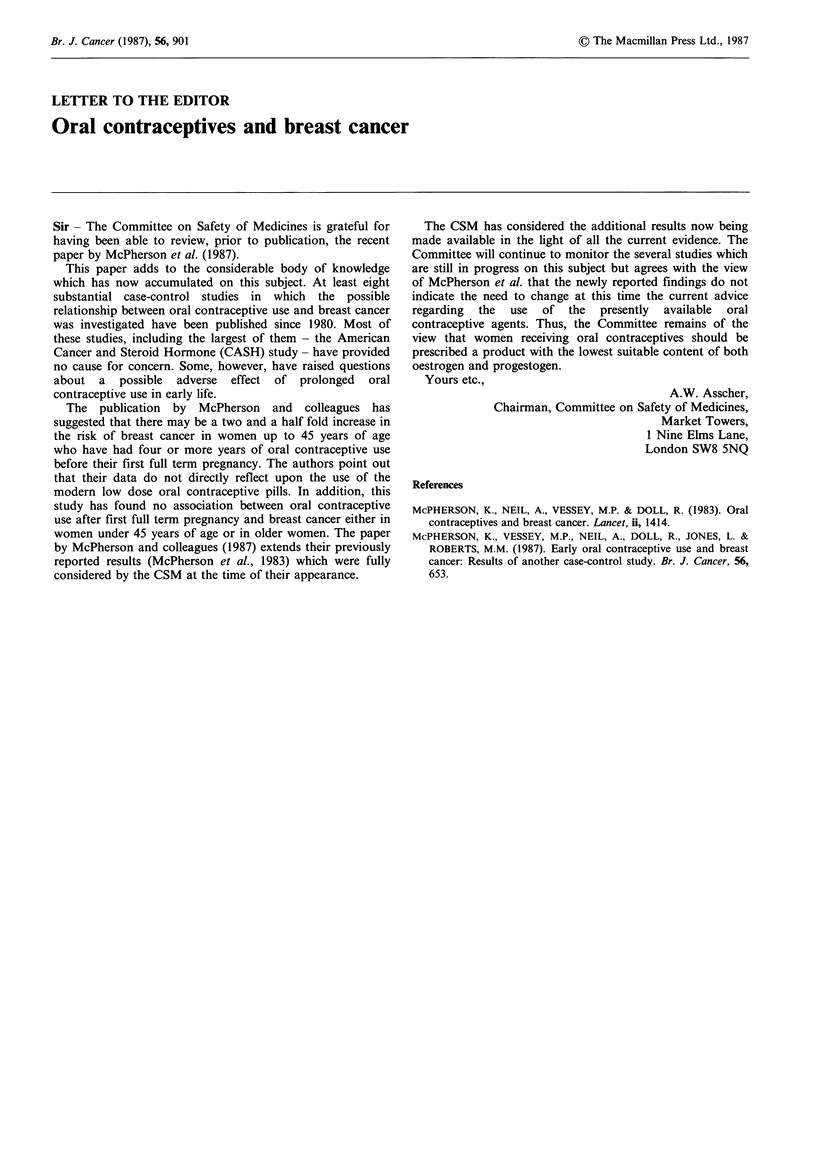# Oral contraceptives and breast cancer.

**DOI:** 10.1038/bjc.1987.308

**Published:** 1987-12

**Authors:** A. W. Asscher


*Br. J. Cancer* (1987), **56**, 901 © The Macmillan Press Ltd., 1987

LETTER TO THE EDITOR

### Oral contraceptives and breast cancer

**Sir** – The Committee on Safety of Medicines is grateful for having been able to review, prior to publication, the recent paper by McPherson *et al.* (1987).

This paper adds to the considerable body of knowledge which has now accumulated on this subject. At least eight substantial case-control studies in which the possible relationship between oral contraceptive use and breast cancer was investigated have been published since 1980. Most of these studies, including the largest of them – the American Cancer and Steroid Hormone (CASH) study – have provided no cause for concern. Some, however, have raised questions about a possible adverse effect of prolonged oral contraceptive use in early life.

The publication by McPherson and colleagues has suggested that there may be a two and a half fold increase in the risk of breast cancer in women up to 45 years of age who have had four or more years of oral contraceptive use before their first full term pregnancy. The authors point out that their data do not directly reflect upon the use of the modern low dose oral contraceptive pills. In addition, this study has found no association between oral contraceptive use after first full term pregnancy and breast cancer either in women under 45 years of age or in older women. The paper by McPherson and colleagues (1987) extends their previously reported results (McPherson *et al.*, 1983) which were fully considered by the CSM at the time of their appearance.

The CSM has considered the additional results now being made available in the light of all the current evidence. The Committee will continue to monitor the several studies which are still in progress on this subject but agrees with the view of McPherson *et al.* that the newly reported findings do not indicate the need to change at this time the current advice regarding the use of the presently available oral contraceptive agents. Thus, the Committee remains of the view that women receiving oral contraceptives should be prescribed a product with the lowest suitable content of both oestrogen and progestogen.

Yours etc.,

A.W. Asscher,
Chairman, Committee on Safety of Medicines,
Market Towers,
1 Nine Elms Lane,
London SW8 5NQ

**References**

McPHERSON, K., NEIL, A., VESSEY, M.P. & DOLL, R. (1983). Oral contraceptives and breast cancer. *Lancet*, **ii**, 1414.

McPHERSON, K., VESSEY, M.P., NEIL, A., DOLL, R., JONES, L. & ROBERTS, M.M. (1987). Early oral contraceptive use and breast cancer: Results of another case-control study. *Br. J. Cancer*, **56**, 653.